# Early Real-World Experience of Switching to Faricimab for Macular Oedema Secondary to Vein Occlusion

**DOI:** 10.3390/life16071183

**Published:** 2026-07-16

**Authors:** Muiz Musadiq, Emer Chang, Abison Logeswaran, Matthew Azzopardi, Mohammed Musadiq, Yu Jeat Chong

**Affiliations:** 1 Department of Ophthalmology, Royal Stoke University Hospital, Stoke-on-Trent ST4 6QG, UK; muiz.musadiq@uhnm.nhs.uk (M.M.); mohammed.musadiq@uhnm.nhs.uk (M.M.); 2 Academic Unit of Ophthalmology, Department of Inflammation and Ageing, School of Infection, Inflammation and Immunology, College of Medicine and Health, University of Birmingham, Birmingham B15 2TT, UK; e.chang@bham.ac.uk; 3 Birmingham and Midland Eye Centre, Sandwell and West Birmingham NHS Trust, Birmingham B18 7QH, UK; 4 Moorfields Eye Hospital, London EC1V 2PD, UK; a.logeswaran@nhs.net (A.L.); matthew.azzopardi.14@um.edu.mt (M.A.)

**Keywords:** retinal vein occlusion, faricimab, anti-VEGF

## Abstract

Aim: To evaluate the real-world effectiveness, durability, and safety of faricimab 6 mg in eyes with treatment-refractory retinal vein occlusion (RVO)-associated macular oedema (MO) in the United Kingdom (UK). Methods: This was a retrospective, single-centre observational study of eyes with RVO that were switched to faricimab after prior treatment with previous anti-vascular endothelial growth factor (anti-VEGF) agents. Baseline demographics, treatment history, pinhole visual acuity (VA), optical coherence tomography (OCT) biomarkers, and injection intervals were recorded. Eyes were treated using a treat-and-extend regimen without a loading phase. Functional, anatomical, durability, and safety outcomes were assessed over follow-up. Results: A total of 22 eyes from 22 patients were included, with a mean (SD) age of 67.9 (11.9) years and a mean (SD) RVO duration of 201.4 (153.1) weeks. Eyes had received a mean (SD) of 20.8 (16.9) prior anti-VEGF injections. The mean (SD) follow-up was 45.7 (15.8) weeks, with a mean (SD) of 5.9 (2.3) faricimab injections. There was no significant change in pinhole VA (53.5 (18.5) vs. 55.0 (18.8) letters, *p* = 0.08). The central subfield thickness (CST) reduced from 407.6 (102.1) to 377.0 (186.5) µm, and the maximum central retinal thickness from 483.6 (103.1) to 442.2 (187.2) µm, although these changes were not statistically significant (*p* > 0.05). The proportion of eyes with subretinal fluid (SRF) decreased from 18.2% to 4.5%, and intraretinal fluid (IRF) from 100% to 81.8%. Injection intervals between the first and second faricimab injections increased significantly from 4.9 (1.4) to 7.7 (3.4) weeks (final injection interval, *p* = 0.004). Six eyes (27.3%) discontinued faricimab, with three (13.6%) requiring an intravitreal dexamethasone implant following a suboptimal response. One eye (4.5%) developed transient intraocular pressure elevation; no cases of intraocular inflammation or endophthalmitis were observed. Conclusions: In this heavily pre-treated, chronic RVO cohort, switching to faricimab without a loading phase resulted in stable visual acuity, modest but non-significant anatomical improvements, and a significant extension in treatment intervals. These findings suggest that faricimab may provide durability benefits and disease stabilisation in treatment-refractory RVO, although functional gains may be limited in chronic disease. Further prospective studies are required to define optimal switching strategies.

## 1. Introduction

Retinal vein occlusions (RVO) represent the second leading cause of retinal vascular disorders [1]. While clinical classification can be challenging, it can be broadly divided into three main types: central RVO (CRVO), branch RVO (BRVO), and hemi RVO (HRVO) [2,3]. Macular oedema (MO) is a major complication in RVO, which can lead to vision loss and legal blindness [4,5]. The pathophysiology of RVO is multifactorial and differs between CRVO and BRVO, with HRVO often considered a subtype of BRVO—common to all RVO types is the upregulation of vascular endothelial growth factor (VEGF), which leads to increased retinal vascular permeability resulting in MO [1,3]. While laser photocoagulation and intravitreal steroids have demonstrated therapeutic benefits in eyes with MO secondary to RVO, anti-vascular endothelial growth factor (anti-VEGF) agents are often still the first-line treatment of choice [6,7].

The efficacy of anti-VEGF in reducing MO and improving visual acuity (VA) has been demonstrated in pivotal clinical trials [8,9,10]. Despite this, many eyes can have variable responses to anti-VEGF therapy in the real world [11,12]. Notably, even within a controlled clinical trial setting such as LEAVO, up to 70% of eyes exhibited persistent or recurrent MO despite being on anti-VEGF treatment for over 100 weeks [13].

Another key mediator implicated in the pathogenesis of RVO is angiopoietin-2 (Ang-2). Patients with RVO have been shown to exhibit some of the highest intravitreal levels of Ang-2 compared with other retinal diseases [14]. Ang-2 acts synergistically with VEGF to drive disease progression, and its upregulation promotes vascular instability, increased permeability, and inflammation [15,16].

The more recent BALATON and COMINO trials evaluated faricimab, a bispecific antibody that exerts its effect through dual inhibition of VEGF-A and Ang-2, in treatment-naïve patients with RVO [17,18]. Results from these studies demonstrated that faricimab was clinically effective, with improvements in VA and reductions in central subfield thickness (CST) from baseline that were sustained through week 72. Notably, treatment durability was enhanced, with more than 45% of patients receiving faricimab at dosing intervals of 12 weeks or longer by week 48 [18]. Subsequently, faricimab was approved by theNational Health Service (NHS) in the United Kingdom (UK) for the treatment of RVO in September 2024 [19].

Although these findings are encouraging, they were derived from rigorously selected, treatment-naïve eyes managed using fixed dosing schedules. It remains uncertain whether comparable outcomes can be achieved in eyes with treatment-resistant MO that have undergone prior intensive anti-VEGF therapy. At present, real-world evidence regarding the use of faricimab in previously treated RVO eyes within the NHS is scarce. This present study seeks to address this evidence gap by assessing the real-world effectiveness, durability, and safety of faricimab in a cohort of eyes with previously treated RVO.

## 2. Methods

This retrospective, real-world observational study was undertaken at a single UK NHS clinical site (Royal Stoke University Hospital, Stoke-on-Trent). This study was registered as an audit with Royal Stoke University Hospital (audit number CA10326) and adhered to the tenets of the Declaration of Helsinki. Patients who were switched to faricimab were identified from our electronic health records. Patient confidentiality was safeguarded throughout the study, and all data were fully anonymised prior to analysis. Patient visits occurred between November 2024 and March 2026, with March 2026 serving as the data cut-off for this interim analysis.

Eyes were eligible for inclusion if they had a diagnosis of RVO and had received prior treatment with more than one anti-VEGF agent and/or dexamethasone implant before being switched to faricimab 6 mg. Only one eye per patient was included, as bilateral switching to faricimab did not occur in this cohort.

No predefined exclusion criteria were applied. However, as per routine clinical practice, eyes with media opacities preventing a reliable optical coherence tomography (OCT) assessment or with advanced disease deemed unsuitable for further anti-VEGF therapy were not selected for treatment switch and were consequently not included in this cohort.

Prior to switching, anti-VEGF therapy had been delivered pragmatically and was predominantly administered on a pro re nata basis rather than a fixed or strict treat-and-extend regimen, with a substantial variation in treatment intensity across the cohort.

### 2.1. Data Collection

Baseline data included patient demographics (age, sex), lens status, pinhole VA, history of prior retinal laser, and anti-VEGF treatment history. The baseline visit was defined as the visit at which the decision to switch to faricimab was made.

OCT parameters included the presence of subretinal fluid (SRF), intraretinal fluid (IRF), ellipsoid zone (EZ) loss within 500 µm of the fovea, CST, maximum central retinal thickness (Cmax), and presence of epiretinal membrane (ERM). Follow-up data included the number of injections, injection intervals, intraocular pressure (IOP) measurements, and both anatomical and functional outcomes.

Final outcomes were defined as measurements obtained at the last available visit.For eyes that discontinued faricimab, outcomes were taken from the final study visit prior to discontinuation after the most recent injection and before any subsequent treatment.

### 2.2. Treatment Protocol

Eyes were switched to faricimab 6 mg without a loading regimen, at the discretion of the clinician. For the purposes of this study, persistent disease activity was defined as ongoing IRF and/or SRF on OCT despite continued anti-VEGF therapy, as distinct from recurrence after a period of quiescence. Eyes were considered treatment-refractory if MO persisted or recurred despite prior anti-VEGF treatment. A pragmatic, disease activity-guided treat-and-extend (T&E) regimen with a treat-to-dry strategy was used. OCT features that included SRF and IRF were assessed qualitatively and with follow-up scans. If SRF or IRF persisted, the injection interval was maintained. In cases of worsening or new disease activity, such as increased SRF/IRF, or haemorrhage, the interval was shortened by two weeks. If fluid improved or reduced, the interval was extended by two weeks. Some eyes were also treated with a combination of macular grid laser or micropulse laser for MO [2,20]. Neovascularisation was treated with focal photocoagulation [2].

Retinal ischaemia was managed with sectoral or scatter photocoagulation, and focal laser was used in selected eyes; these were often administered prior to switching to faricimab. No macular grid or micropulse laser was administered during the study period.

### 2.3. OCT Imaging

Macular OCT was performed using the Spectralis OCT system (Heidelberg Engineering, Heidelberg, Germany), with a 19-line sectional scanning protocol at a resolution of 200 µm. The infrared image was acquired at 1536 × 1536 pixels (high resolution) over a 30-degree field (8.7 mm), and the OCT scan was acquired at 1024 × 496 pixels over a 20-degree field (5.8 mm). Retinal layer segmentation was reviewed by a senior consultant ophthalmologist (MM) and manually corrected when necessary.

### 2.4. Statistical Analysis

Continuous variables are presented as mean and standard deviation (SD), while categorical variables are reported as frequency and percentage. Normality was assessed using the Shapiro–Wilk test. Normally distributed paired data were compared using the paired *t*-test. Non-normally distributed paired data were compared using the Wilcoxon signed rank test. Paired proportions were compared using McNemar’s test.

## 3. Results

A total of 22 eyes from 22 patients were included. The mean (SD) age of patients was 67.9 (11.9) years. 13 (59.1%) patients were male; 18 patients had a diagnosis of hypertension; 7 (31.8%) were diabetic, and 6 (27.3%) were smokers. 2 (9.1%) eyes had a pre-existing diagnosis of glaucoma. None of the patients had a diagnosis of RVO in the fellow eye.

In terms of diagnosis, nine (40.9%) had CRVO, while 12 (54.5%) had BRVO and 1 (4.5%) had HRVO. Regarding prior retinal laser, 9 eyes (40.9%) had sectoral/scatter photocoagulation for retinal ischaemia and 2 eyes (9.1%) had focal laser. During faricimab follow-up, a further 2 eyes received adjunctive laser (focal laser in one eye and sectoral photocoagulation in one eye).

The mean (SD) baseline pinhole VA was 53.5 (18.5) letters (Snellen equivalent 6/24). Less than one third of eyes (6; 27.3%) recorded ≥70 letters (Snellen equivalent 6/12). At the time of switch, the mean (SD) baseline CST was 407.6 (102.1) μm, with a mean (SD) Cmax of 483.6 (103.1) μm. 4 (18.2%) eyes had SRF, while all eyes (100%) had IRF. The presence of EZ loss and ERM was 12 (54.5%) and 3 (13.6%), respectively.

The mean (SD) time from initial diagnosis to faricimab switch was 201.4 (153.1) weeks. The mean (SD) number of previous anti-VEGF injections was 20.8 (16.9) with the mean (SD) time from last anti-VEGF to baseline visit being 12.5 (21.1) weeks. 4 (18.2%) eyes had a previous intravitreal dexamethasone 700 µg implant: of these eyes, the mean (SD) number of previous anti-VEGF injections was higher than eyes without intravitreal dexamethasone, but this was not statistically significant (35.0 (19.5) versus 17.6 (15.1), *p* = 0.07).

The majority of eyes (20; 90.9%) were switched due to persistent disease activity. The remaining eyes were switched either to increase injection interval (1; 4.5%), or due to new disease activity (1; 4.5%).

These baseline characteristics are summarised in Table 1.

### 3.1. Treatment Summary

Following switching to faricimab, the mean (SD) number of injections per eye was 5.9 (2.3). The mean (SD) number of visits was 8.6 (3.1), with the mean (SD) follow-up duration of 45.7 (15.8) weeks.

The mean (SD) injection interval at the final visit was 7.7 (3.4) weeks, compared to the mean (SD) injection interval between the first and second faricimab injections of 4.9 (1.4) weeks, *p* = 0.004.

6 eyes (27.3%) discontinued faricimab during follow-up, after a variable number of injections. 3 eyes (13.6%) were switched to an intravitreal dexamethasone implant following a suboptimal response, having received six, three and five faricimab injections, respectively; one eye discontinued after two injections, owing to poor prognosis with foveal-involving atrophy; one eye discontinued after two injections, having achieved disease stability with resolution of intraretinal and subretinal fluid; and one eye was lost to follow-up after three injections.

**Figure 1:**
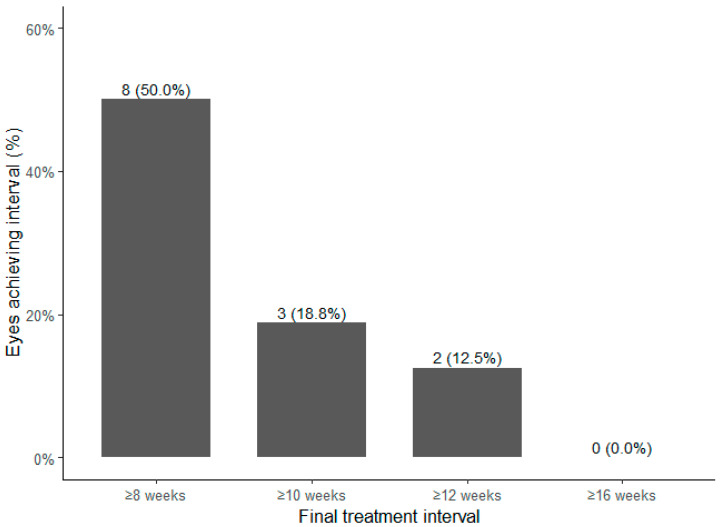
Final treatment interval of eyes that remained on faricimab (*n* = 16).

Among the 16 eyes that remained on faricimab treatment, eight (50%) achieved a final treatment interval of ≥8 weeks, three (18.8%) achieved ≥10 weeks, and two (12.5%) achieved ≥12 weeks. No eyes achieved a final treatment interval of ≥16 weeks (Figure 1).

### 3.2. Functional and Anatomical Outcomes

At the final visit, the mean (SD) pinhole VA was 55.0 (18.8) letters, with no difference compared to baseline, *p* = 0.08. There were seven (31.8%) eyes with ≥70 letters, which was not statistically significant compared to baseline, *p* > 0.99.

**Figure 2:**
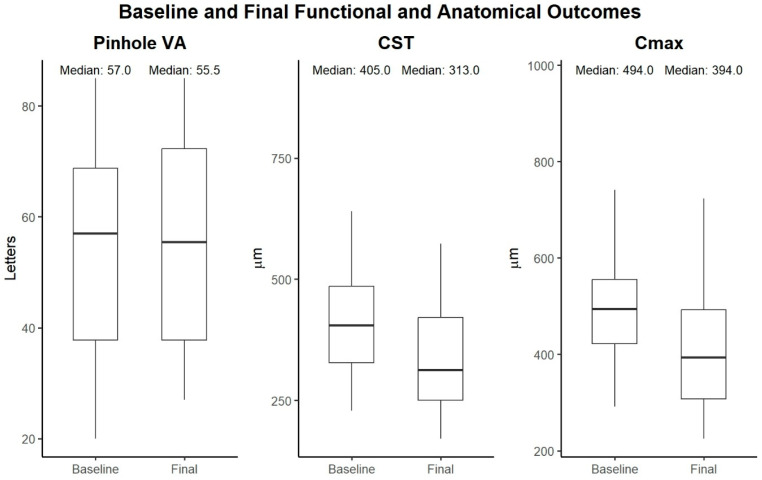
Baseline and final functional and anatomical outcomes. VA, visual acuity; CST, central subfield thickness; Cmax, maximum central retinal thickness.

At the final visit, one (4.5%) eye had SRF and eighteen (81.8%) eyes had IRF—this was a decrease compared to baseline (*p* = 0.37 for SRF comparison; no statistical test was performed for IRF due to a lack of variability at baseline as all eyes had IRF). There were reductions in both CST 377.0 (186.5) μm and Cmax 442.2(187.2) μm compared to baseline; however, neither was significant (*p* > 0.05 for both). Baseline and final functional and anatomical outcomes are illustrated in Figure 2. These treatment outcomes are summarised in Table 2. Further details of paired continuous outcomes are summarised in Table 3.

### 3.3. Safety

One eye (4.5%) required IOP-lowering therapy (apraclonidine) following an intraocular pressure elevation to 50 mmHg at the fourth faricimab injection. The patient continued treatment, receiving seven further injections without recurrence of IOP elevation. No eyes developed uveitis or endophthalmitis. No neovascular complications were observed during follow-up.

## 4. Discussion

We present a real-world evaluation of faricimab 6 mg in a UK NHS cohort of heavily pre-treated, treatment-refractory RVO eyes. Several key findings emerge from this study. Firstly, VA remained stable without significant improvement following switching treatments. Secondly, while there was a trend towards anatomical improvement, including reductions in CST and Cmax and a decrease in the proportion of eyes with retinal fluid, these changes did not reach statistical significance. Thirdly, treatment durability improved, with a significant extension in injection intervals. Finally, faricimab demonstrated a favourable safety profile, with minimal adverse events.

The limited functional improvement observed is likely attributable to the chronicity and refractory nature of the cohort. Eyes had a mean disease duration of 201.4 weeks (approximately 3.9 years) and had received a mean of 20.8 prior anti-VEGF injections, reflecting longstanding disease with substantial prior treatment exposure. Baseline OCT findings further support this, with EZ loss present in more than half of the eyes (54.5%), indicating photoreceptor disruption. In addition, all eyes exhibited intraretinal IRF at baseline, with a mean CST of 407.6 μm, suggesting persistent oedema despite intensive therapy. In this context, the absence of visual gain is not unexpected, as chronic retinal damage and photoreceptor loss may limit the potential for functional recovery even when anatomical improvements occur [21].

Although reductions in CST and retinal fluid were not statistically significant, the direction of change suggests a biological effect of faricimab. The proportion of eyes with SRF decreased from 18.2% at baseline to 4.5% at final follow-up, and IRF was reduced from 100% to 81.8%, indicating a partial anatomical response. Persistent oedema in this cohort may reflect not only ongoing VEGF-driven permeability but also chronic microvascular dysfunction and oxidative stress-mediated injury, which are increasingly recognised as key contributors to RVO pathophysiology. Oxidative stress interacts with hypoxia-driven pathways to promote VEGF expression, inflammation, and blood–retinal barrier breakdown, contributing to ongoing neurovascular damage [22].

In our cohort, VA remained stable, which is consistent with a ceiling effect in chronic RVO. VA gains are known to usually occur early after treatment initiation, as demonstrated in the global phase 3 BALATON and COMINO trials, where both functional and anatomical improvements were observed following initial faricimab treatment and were sustained up to 72 weeks, including in the faricimab switch cohort [17,18]. In these randomised controlled trials, treatment-naïve eyes with RVO were initially randomised to receive 4-weekly faricimab 6 mg or aflibercept 2 mg for 20 weeks, followed by faricimab from week 24 to 72 using a modified T&E regimen guided by CST and VA [23]. The key difference between the BALATON/COMINO cohorts and our study population lies in disease chronicity: eyes in the trials were treatment-naïve with recent onset RVO, whereas our cohort comprised treatment-refractory eyes with chronic disease prior to switching.

There remains ongoing debate regarding whether patients switched to faricimab should receive a loading course to maximise the effect of Ang-2 blockade or continue at their existing treatment interval without loading [24]. Our findings suggest that a loading phase may be required to optimise the treatment response in refractory RVO. Both our study and a retrospective analysis by Hirakata et al. found that switching to faricimab without loading resulted in anatomical improvement without a corresponding functional gain in VA [24]. In contrast, Hafner et al. demonstrated sustained anatomical and functional improvement at nine months following a loading phase of four consecutive monthly faricimab injections in treatment-refractory RVO with a similar disease duration [25,26]. This likely reflects the temporal dynamics of dual VEGF and Ang-2 inhibition: VEGF blockade produces a relatively rapid reduction in CST, whereas vascular stabilisation through the Tie2 pathway requires prolonged and sustained Ang-2 suppression [16,27]. Prospective studies directly comparing loading versus non-loading switch strategies in refractory RVO cohorts are therefore warranted.

Notably, even with a loading regimen, 20% of patients in the Hafner et al. cohort could not be extended beyond monthly dosing at nine months [26]. This may reflect irreversible vascular damage in chronic RVO and highlights the importance of setting realistic treatment goals. In such cases, accepting residual fluid may be appropriate if vision is stable and longer treatment intervals are preferred by the patient.

The reasons for treatment discontinuation were heterogeneous and included a suboptimal response requiring corticosteroid therapy, poor visual prognosis due to foveal-involving atrophy, achievement of disease stability, and loss to follow-up. This variability highlights the clinical heterogeneity of refractory RVO and suggests that mechanisms beyond VEGF-mediated vascular permeability, including inflammatory pathways, may contribute to persistent disease activity in some eyes. Eyes that discontinued faricimab tended to have a higher prevalence of ellipsoid zone loss (83.3% vs. 43.8%) and lower baseline central subfield and maximum macular thicknesses than those that continued (Table 4), suggesting more advanced structural damage and less active oedema—a formal comparison was not undertaken given the small number of discontinued eyes.

It is also notable that 27.3% of eyes discontinued faricimab, with 13.6% requiring subsequent treatment with an intravitreal dexamethasone implant following a suboptimal response. This highlights the heterogeneity of the treatment response in refractory RVO and suggests that inflammatory mechanisms may play a role in a subset of eyes. Intravitreal steroids have been shown to be effective in reducing macular MO in RVO, particularly in eyes with an incomplete response to anti-VEGF therapy [28]. Although pars plana vitrectomy with internal limiting membrane peeling has been reported to improve visual acuity in RVO-associated MO, including CRVO [29], it is not part of current UK guideline-based management [2], which typically reserves surgery for complications or a visually significant tractional epiretinal membrane. In our cohort, ERM was present in only three eyes (13.6%); none had a tractional component warranting surgery.

In terms of safety, faricimab was well tolerated. Only one eye (4.5%) developed an intraocular pressure elevation, which was successfully managed with topical therapy without recurrence. No cases of intraocular inflammation or endophthalmitis were observed. These findings are consistent with the favourable safety profile reported in clinical trials [18]. The absence of significant inflammatory events in this cohort is reassuring, particularly given previous reports of rare intraocular inflammation associated with faricimab [30].

This study has several limitations. Firstly, the small sample size increased the risk of Type II errors. Additionally, heterogeneity in the RVO subtype, prior treatment exposure, and adjunctive therapies may have influenced outcomes. A direct comparison with the pre-switch anti-VEGF treatment interval was not possible, as the switch visit frequently coincided with the last anti-VEGF injection and pre-switch injection intervals were not reliably available; the interval extension reported here therefore reflects change during faricimab treatment rather than a comparison with prior therapy. Furthermore, a baseline retinal perfusion status was not systematically assessed, as OCT-angiography and fluorescein angiography were not routinely performed at the time of switching. Finally, the relatively short follow-up duration (mean 45.7 weeks) may not fully capture the long-term effects of faricimab. Future studies with larger cohorts, a longer follow-up, and stratification by ischemic status are needed to better define treatment responses [31].

## 5. Conclusions

In this real-world UK NHS cohort of heavily pre-treated RVO eyes, switching to faricimab 6 mg without a loading phase resulted in stable visual acuity, modest but non-significant anatomical improvements, and a significant extension in treatment intervals. These findings suggest that while faricimab may not reverse established structural damage in chronic RVO, it may provide meaningful durability benefits and disease stabilisation in treatment-refractory eyes. Further prospective studies are required to optimise switching strategies and identify patients who are most likely to benefit from dual VEGF-A and Ang-2 inhibition. 

## Figures and Tables

**Table 1:** Baseline characteristics.

Patient Level (*n* = 22)		
Age, years	Mean (SD)	67.9 (11.9)
Male	*n*%	13 (59.1)
Hypertension	*n*%	18 (81.8)
Diabetic	*n*%	7 (31.8)
Smoking	*n*%	6 (27.3)
RVO in fellow eye	*n%*	0 (0.0)
**Eye level (*n* = 22)**		
Pre-existing glaucoma	*n*%	2 (9.1)
CRVO	*n*%	9 (40.9)
BRVO	*n*%	12 (54.5)
HRVO	*n*%	1 (4.5)
Previous focal laser	*n*%	2 (9.1)
Previous sectoral laser (PRP for ischaemia)	*n*%	9 (40.9)
Pseudophakic	*n*%	7 (31.8)
Pinhole VA, letters	Mean (SD)	53.5 (18.5)
≥70 letters	*n*%	6 (27.3)
Presence of SRF	*n*%	4 (18.2)
Presence of IRF	*n*%	22 (100.0)
CST, μm	Mean (SD)	407.6 (102.1)
Cmax, μm	Mean (SD)	483.6 (103.1)
Presence of EZ loss	*n*%	12 (54.5)
Presence of ERM	*n*%	3 (13.6)
Time from initial diagnosis to switch, weeks	Mean (SD)	201.4 (153.1)
Previous dexamethasone implant	*n*%	4 (18.2)
**Previous anti-VEGF treatment**		
Number of previous injections	Mean (SD)	20.8 (16.9)
Time from last anti-VEGF to baseline, weeks	Mean (SD)	12.5 (21.1)
**Last treatment before switch to faricimab**		
Aflibercept 2 mg	*n*%	20 (91.0)
Ranibizumab	*n*%	1 (4.5)
Dexamethasone implant	*n*%	1 (4.5)
**Reason for switching**		
Increase interval	*n*%	1 (4.5)
Persistent disease activity	*n*%	20 (90.9)
New disease activity	*n*%	1 (4.5)

RVO, retinal vein occlusion; CRVO, central retinal vein occlusion; BRVO, branch retinal vein occlusion; HRVO, hemi retinal vein occlusion; VA, visual acuity; PRP, pan-retinal photocoagulation; IRF, intraretinal fluid; SRF, subretinal fluid; CST, central subfield thickness; EZ, ellipsoid zone; ERM, epiretinal membrane.

**Table 2:** Treatment outcomes.

Eye Level (*n* = 22)		
Number of faricimab injections following switch	Mean (SD)	5.9 (2.3)
Number of follow-up visits	Mean (SD)	8.6 (3.1)
Injection interval at final visit, weeks	Mean (SD)	7.7 (3.4)
Eyes that discontinued faricimab	*n* (%)	6 (27.3)
Pinhole VA at final visit, letters	Mean (SD)	55.0 (18.8)
≥70 letters at final visit	*n* (%)	7 (31.8)
SRF present at final visit	*n* (%)	1 (4.5)
IRF present at final visit	*n* (%)	18 (81.8)
CST at final visit, μm	Mean (SD)	377.0 (186.5)
Cmax at final visit, μm	Mean (SD)	442.2 (187.2)

VA, visual acuity; SRF, subretinal fluid; IRF, intraretinal fluid; CST, central subfield thickness; Cmax, maximum central retinal thickness.

**Table 3:** Outcomes following the switch to faricimab.

Outcome	Baseline	Final	Mean Change (95% CI)	Median Change (IQR)	*p*-Value
Primary analysis, all eyes (*n* = 22)					
Pinhole VA, letters	53.5	55.0	1.5 (−3.0 to 6.1)	3.0 (0.0 to 8.8)	0.08
CST, μm	407.6	377.0	−30.6 (−116.1 to 54.9)	−54.0 (−138.2 to 42.8)	0.24
Cmax, μm	483.6	442.2	−41.4 (−123.7 to 40.9)	−88.0 (−158.5 to 61.0)	0.18
Injection interval, weeks	4.9	7.7	2.8 (1.0 to 4.5)	2.2 (0.0 to 4.3)	<0.01
Sensitivity analysis, eyes continued on faricimab (*n* = 16)					
Pinhole VA, letters	51.8	54.1	2.3 (−3.8 to 8.5)	3.0 (−0.2 to 10.0)	0.05
CST, μm	427.2	371.5	−55.8 (−141.7 to 30.2)	−77.5 (−145.0 to −1.8)	0.17
Cmax, μm	502.9	436.1	−66.8 (−151.9 to 18.2)	−90.5 (−169.0 to 13.0)	0.12
Injection interval, weeks	4.9	7.5	2.6 (0.7 to 4.4)	2.9 (1.1 to 4.1)	0.01

VA, visual acuity; CST, central subfield thickness; IQR, interquartile range; CI, confidence interval; CST, central subfield thickness; Cmax, maximum central retinal thickness.

**Table 4:** Baseline characteristics of eyes that continued faricimab versus eyes that discontinued.

Characteristic	Continued (*n* = 16)	Discontinued (*n* = 6)
RVO subtype		
CRVO, *n* (%)	6 (37.5)	3 (50.0)
BRVO, *n* (%)	9 (56.3)	3 (50.0)
HRVO, *n* (%)	1 (6.3)	0 (0)
Disease duration, weeks, median (IQR)	149.5 (90.0–269.2)	173.5 (67.0–318.2)
Baseline VA, letters, median (IQR)	57.0 (33.0–64.0)	56.5 (42.0–71.8)
Baseline CST, µm, median (IQR)	419.0 (352.0–509.0)	364.0 (299.8–420.8)
Baseline Cmax, µm, median (IQR)	509.0 (426.8–571.0)	471.0 (377.2–499.5)
EZ loss, *n* (%)	7 (43.8)	5 (83.3)
ERM, *n* (%)	3 (18.8)	0 (0)
Previous dexamethasone implant, n (%)	2 (12.5)	2 (33.3)
Prior anti-VEGF injections, median (IQR)	17.0 (6.8–31.0)	10.0 (7.8–30.2)

RVO, retinal vein occlusion; CRVO, central retinal vein occlusion; BRVO, branch retinal vein occlusion; HRVO, hemi retinal vein occlusion; VA, visual acuity; CST, central subfield thickness; Cmax, maximum central retinal thickness; EZ, ellipsoid zone; ERM, epiretinal membrane. Given the small number of discontinued eyes, comparisons are descriptive with no formal statistical testing.

## Data Availability

Data is available from the corresponding author on reasonable request.
